# Circulating Tumor Necrosis Factor α Receptors Predict the Outcomes of Human IgA Nephropathy: A Prospective Cohort Study

**DOI:** 10.1371/journal.pone.0132826

**Published:** 2015-07-15

**Authors:** Yun Jung Oh, Jung Nam An, Clara Tammy Kim, Seung Hee Yang, Hajeong Lee, Dong Ki Kim, Kwon Wook Joo, Jin Ho Paik, Shin-Wook Kang, Jung Tak Park, Chun Soo Lim, Yon Su Kim, Jung Pyo Lee

**Affiliations:** 1 Department of Internal Medicine, Cheju Halla General Hospital, Jeju, Korea; 2 Department of Internal Medicine, Seoul National University Boramae Medical Center, Seoul, Korea; 3 Department of Internal Medicine, Seoul National University College of Medicine, Seoul, Korea; 4 Graduate School of Public Health, Seoul National University, Seoul, Korea; 5 Kidney Research Institute, Seoul National University, Seoul, Korea; 6 Department of Pathology, Seoul National University College of Medicine, Seoul, Korea; 7 Department of Internal Medicine, Severance Biomedical Science Institute, Yonsei University College of Medicine, Seoul, Korea; University of Central Florida, UNITED STATES

## Abstract

The circulating tumor necrosis factor receptors (TNFRs) could predict the long-term renal outcome in diabetes, but the role of circulating TNFRs in other chronic kidney disease has not been reported. Here, we investigated the correlation between circulating TNFRs and renal histologic findings on kidney biopsy in IgA nephropathy (IgAN) and assessed the notion that the circulating TNFRs could predict the clinical outcome. 347 consecutive biopsy-proven IgAN patients between 2006 and 2012 were prospectively enrolled. Concentrations of circulating TNFRs were measured using serum samples stored at the time of biopsy. The primary clinical endpoint was the decline of estimated glomerular filtration rate (eGFR; ≥ 30% decline compared to baseline). Mean eGFR decreased and proteinuria worsened proportionally as circulating TNFR1 and TNFR2 increased (*P* < 0.001). Tubulointerstitial lesions such as interstitial fibrosis and tubular atrophy were significantly more severe as concentrations of circulating TNFRs increased, regardless of eGFR levels. The risks of reaching the primary endpoint were significantly higher in the highest quartile of TNFRs compared with other quartiles by the Cox proportional hazards model (TNFR1; hazard ratio 7.48, *P* < 0.001, TNFR2; hazard ratio 2.51, *P* = 0.021). In stratified analysis according to initial renal function classified by the eGFR levels of 60 mL/min/1.73 m^2^, TNFR1 and TNFR2 were significant predictors of renal progression in both subgroups. In conclusion, circulating TNFRs reflect the histology and clinical severity of IgAN. Moreover, elevated concentrations of circulating TNFRs at baseline are early biomarkers for subsequent renal progression in IgAN patients.

## Introduction

IgA nephropathy (IgAN) is the most common form of glomerulonephritis (GN) worldwide, especially in Asia [1,2]. IgAN is a clinically heterogeneous disease, and it has been considered benign because of its indolent course. However, 30–50% of patients with IgAN eventually progress to end-stage renal disease (ESRD) within 30 years of diagnosis [1,3–5], and IgAN is also related to mortality not derived from ESRD [5].

Several prior studies have attempted to predict long-term prognosis at the time of initial diagnosis. Renal insufficiency [3,6,7], hypertension [7–9], persistent or severe proteinuria [6,8–10], and certain histological features [3,8,9,11] have been identified as the important risk factors for renal progression. However, there is no secure biomarker that can indicate the severity and predict the long-term prognosis of IgAN, and reflect the responsiveness of specific treatment.

Tumor necrosis factor-α (TNFα) is a key mediator with proinflammatory and immunoregulatory properties. TNFα is mediated via TNF receptor 1 (TNFR1) and TNF receptor 2 (TNFR2), which are membrane bound and soluble in plasma [12]. Elevated serum concentrations of circulating TNFRs have been found in chronic kidney disease [13–17], and recent studies reported a strong correlation with early and late renal progression in type 1 and 2 diabetes [18,19]. In addition, TNFα pathway markers were up-regulated in lupus nephritis along with elevated urinary TNFR1 excretion [20,21]. Urinary TNFR1 excretion positively correlated with serum creatinine and proteinuria in primary GN [22]. However, the relationship of circulating TNFRs at diagnosis with clinical manifestations and prognosis has not been established in IgAN.

We therefore designed this study to identify the relationships between circulating TNFRs and clinical characteristics or pathologic findings and to investigate the correlation of circulating TNFRs at baseline with clinical outcome.

## Materials and Methods

### Ethics statement

This study was approved by the institutional review boards of Seoul National University Hospital and Yonsei University Medical Center in Seoul, Korea (H-1207-072-418). All clinical investigations were conducted in accordance with the guidelines of the 2013 Declaration of Helsinki. Written informed consents were obtained from all participants.

### Patients and serum samples

A total of 347 patients with newly diagnosed, biopsy-proven primary IgAN between 2006 and 2012 were prospectively recruited. IgAN was diagnosed by mesangial depositions of IgA on immunofluorescence microscopy and electron-dense deposits in the mesangium on electron microscopy. Blood specimens collected at the time of kidney biopsy from all study patients were immediately cooled and centrifuged at 3000 rpm for 10 minutes, and serum samples were stored at -70°C until tested.

### Clinical data

Patient demographics and clinical parameters including age, sex, body mass index, blood pressure (BP), blood chemistry, and degree of daily proteinuria, were collected at the time of kidney biopsy. Blood chemistry test included serum creatinine, albumin, uric acid, and IgA. Estimated glomerular filtration rate (eGFR) was calculated using isotope dilution mass spectrometry (IDMS)-traceable Modification of Diet in Renal Disease (MDRD) equation [23]. The urine protein-creatinine ratio (uPCR) in a random urine sample was used to assess the degree of proteinuria. Serial changes in renal function and the degree of proteinuria were recorded during the follow-up period. Primary outcome was defined as a decline of 30% or more in eGFR levels compared with the baseline values.

Medication history, including the use of renin-angiotensin system (RAS) blockers such as angiotensin-converting enzyme inhibitors and angiotensin II receptor blockers, statins, and immunosuppressives (IS) within 6 months of kidney biopsy and during the follow-up period, was recorded. RAS blockers were prescribed to most of the patients who had uPCR ≥ 0.5 even before a kidney biopsy. The kidney biopsy was generally performed in the patients with uncontrolled proteinuria or BP in spite of the treatment with RAS blockers. However, according to the clinical judgment of the nephrologists, it would often be performed regardless of the treatment effects of RAS blockers. In addition, some patients visited our tertiary referral hospitals already taking the RAS blockers. Meanwhile, the patients who had a proteinuria greater than 1g or impaired renal function usually received immunosuppressive (IS) treatment within 2 to 4 weeks after the kidney biopsy. Steroid drugs were mainstay of the treatment. None of the study patients had been received IS treatment before a renal histological confirmation.

### Renal histopathology

All kidney biopsy specimens were obtained by percutaneous kidney biopsy, and examined by light-, immunofluorescence-, and electron microscopy. All biopsy slides were evaluated by an experienced renal pathologist. The number of glomeruli, global sclerosis, and crescentic lesions were counted. The severity of glomerular sclerosis and crescent formation were classified as normal mild (< 25%), moderate (25–49%), and severe (≥ 50%) based on percentages. For tubulointerstitial lesions, interstitial inflammation, interstitial fibrosis, and tubular atrophy were graded semi-quantitatively (normal to severe). In addition, mesangial cells were counted per mesangial area, and mesangial hypercellularity was graded [24,25].

### Measurement of TNFRs

Circulating TNFR1 and TNFR2, both free and bound with TNFα were measured by enzyme-linked immunosorbent assay (ELISA; R&D systems, Minneapolis, MN, USA) according to the manufacturer’s protocol. All measurements were performed in blind manner and in duplicate.

### Statistical analysis

Categorical variables described as frequencies and proportions were compared using chi-squared tests; after test of normality, non-normally distributed variables were shown as the median (25^th^, 75^th^ percentiles) and compared with the Kruskal-Wallis test. Comparisons among groups (first to fourth quartile of TNFRs) were performed using Dunn’s method for multiple comparisons for non-parametric variables. Event free survival curves were derived from the Kaplan-Meier method and differences between the two curves were tested by the log-rank test. The Cox proportional hazards model was used to identify independent predictors for the development of primary endpoint. A correlation analysis was conducted in order to avoid multi-collinearity; only one variable in highly correlated variable sets was selected for multivariate analysis. Statistically significant covariables from univariate analysis and clinically important covariables were included in the final multivariate Cox proportional hazard regression analysis, and then conducted in a backward stepwise manner. *P*-values were two-tailed and were determined to be statistically significant when less than 0.05. Statistical analysis was performed using SPSS software (version 19.0, Chicago, IL, USA).

## Results

### Baseline characteristics

Table 1 shows the clinical characteristics of the study population (n = 347) at the time of kidney biopsy. The median age was 37 years and 42.7% were men. Median level of systolic BP, uPCR, and baseline eGFR was 124 mmHg, 0.91 g/g, and 74.2 mL/min/1.73 m^2^, respectively. A total of 255 (73.5%) patients were treated with RAS blockers at the time of kidney biopsy and 43 (12.4%) received immunosuppressive therapy within 2 to 4 weeks after kidney biopsy.

**Table 1:** Baseline characteristics of the study group with IgAN.

	Total (n = 347)
**Age (years)**	37 (29, 48)
**Male (n/%)**	148/42.7%
**Smoker (n/%)**	39/11.2%
**Body mass index (kg/m** ^**2**^ **)**	22.6 (20.5, 24.8)
**Systolic blood pressure (mmHg)**	124 (111, 140)
**Diastolic blood pressure (mmHg)**	80 (70, 90)
**Microscopic hematuria (n/%)**	311/89.6%
**UPCR (g/g)**	0.91 (0.43, 1.97)
**Serum creatinine (mg/dL)**	1.0 (0.8, 1.3)
**eGFR (mL/min/1.73 m** ^**2**^ **)**	74.2 (54.2, 90.8)
**Serum albumin (mg/dL)**	4.0 (3.7, 4.3)
**Serum total cholesterol (mg/dL)**	180 (156, 207)
**Serum IgA (mg/dL)**	283.0 (222.3, 358.5)
**Uric acid (mg/dL)**	5.5 (4.4, 6.9)
**Medical treatment (n/%)**	
** RAS blockers**	255/73.5%
** Statin**	81/23.3%
** Immunosuppressant**	43/12.4%

Data are presented as a number (percent) or a median (25^th^, 75^th^ percentiles). eGFR, estimated glomerular filtration rate; IgAN, IgA nephropathy; RAS, renin-angiotensin system; UPCR, urine protein-creatinine ratio.

### The correlation between clinical parameters and circulating TNFRs

**Fig 1:**
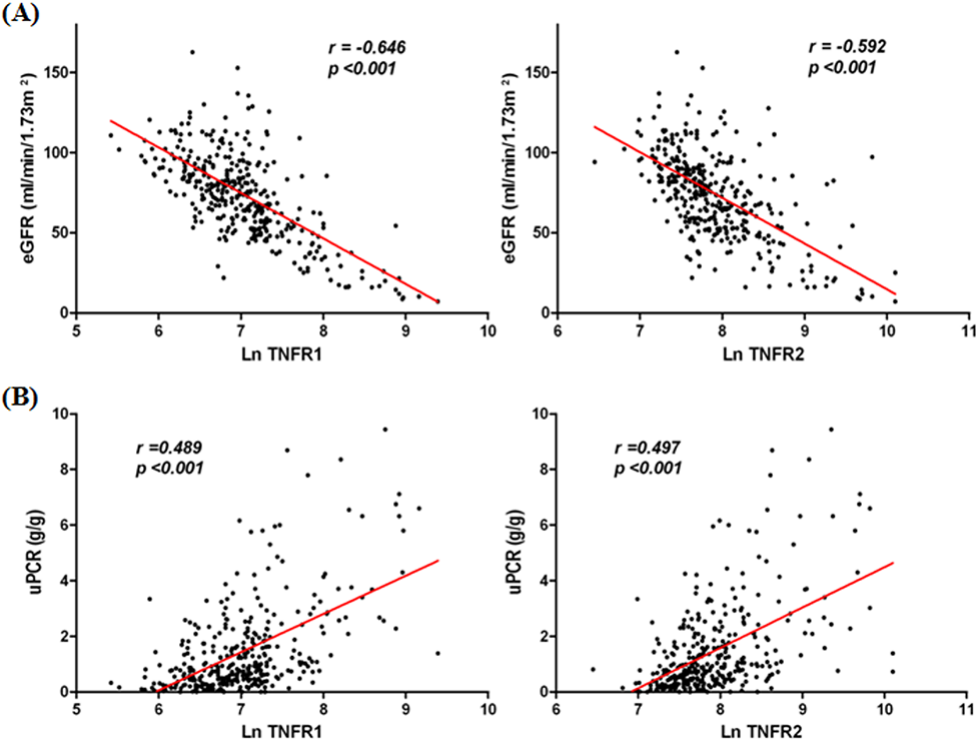
Correlation of circulating TNFRs with eGFR (A) and uPCR (B) at the time of kidney biopsy Log-transformed circulating TNFR1 and TNFR2 were correlated negatively with eGFR (A) and positively with uPCR (B), respectively. eGFR, estimated glomerular filtration rate; TNFR, tumor necrosis factor receptors; uPCR, urine protein-creatinine ratio.

To identify the relationship between circulating TNFRs and several clinical parameters, we divided the patients into quartile based on circulating TNFR concentrations. Higher concentrations of circulating TNFR1 and TNFR2 were significantly associated with lower eGFR and higher uPCR levels (Table 2). Stepwise increases in uric acid levels and decrease in serum albumin levels also corresponded with TNFR quartiles. However, there was no significant correlation of SBP and serum IgA levels with TNFRs. These correlations were also observed when TNFRs were used as continuous variables (Table 3, Fig 1). There was a significant positive correlation between cTNFR1 and cTNFR2 (*r* = 0.75) and eGFR or uPCR was also strongly correlated with cTNFRs.

**Table 2:** Clinical parameters according to TNFR1 and TNFR2 concentrations at the time of kidney biopsy.

	**TNFR1 Q1**	**TNFR1 Q2**	**TNFR1 Q3**	**TNFR1 Q4**	***P*** ^d^
**Age (years)**	32 (24, 42)	37 (27, 47)	37 (29, 49)	45 (34, 55)^a^	<0.001
**Body mass index (kg/m** ^**2**^ **)**	21.2 (19.5, 23.7)	22.5 (20.3, 24.5)	23.4 (20.9, 25.1) ^b^	23.4 (20.8, 25.4) ^b^	0.003
**Systolic BP (mmHg)**	122 (110, 135)	120 (114, 140)	124 (112, 136)	130 (117, 142)	0.053
**UPCR (g/g)**	0.49 (0.21, 0.99)	0.69 (0.40, 1.45)	0.93 (0.47, 1.82) ^b^	2.28 (1.05, 3.76) ^a^	<0.001
**Serum creatinine (mg/dL)**	0.80 (0.70, 1.00)	0.90 (0.80, 1.07)	1.06 (0.87, 1.23)^c^	1.46 (1.15, 2.12) ^a^	<0.001
**eGFR (mL/min/1.73 m** ^**2**^ **)**	90.1 (81.9, 102.0)	78.7 (67.1, 92.0)^b^	67.3 (56.9, 86.0) ^b^	45.6 (27.3, 58.6) ^a^	<0.001
**Serum albumin (mg/dL)**	4.4 (4.0, 4.5)	4.1 (3.8, 4.3) ^b^	4.0 (3.8, 4.3) ^c^	3.6 (3.2, 3.9) ^a^	<0.001
**Serum uric acid (mg/dL)**	4.7 (3.8, 5.7)	4.8 (4.2, 5.8)	5.8 (5.1, 7.0) ^c^	7.0 (5.8, 8.2) ^a^	<0.001
**Serum IgA (mg/dL)**	276.0 (233.5, 367.8)	277.0 (220.8, 366.5)	287.0 (236.5, 361.0)	283.0 (202.0, 346.0)	0.727
	**TNFR2 Q1**	**TNFR2 Q2**	**TNFR2 Q3**	**TNFR2 Q4**	***P*** ^d^
**Age (years)**	30 (20, 39)	37 (26, 47) ^b^	39 (31, 51) ^b^	45 (34, 57) ^c^	<0.001
**Body mass index (kg/m** ^**2**^ **)**	21.5 (19.6, 24.7)	22.3 (20.5, 24.4)	23.6 (21.2, 25.2) ^b^	23.1 (20.8, 24.5)	0.041
**Systolic BP (mmHg)**	125 (110, 136)	120 (110, 139)	124 (120, 140)	130 (115, 140)	0.239
**UPCR (g/g)**	0.46 (0.17, 0.81)	0.78 (0.42, 1.58) ^b^	1.13 (0.48, 2.05) ^b^	2.11 (0.94, 3.70) ^a^	<0.001
**Serum creatinine (mg/dL)**	0.81 (0.70, 0.98)	0.94 (0.80, 1.09) ^b^	1.08 (0.87, 1.25) ^b^	1.30 (1.09, 2.10) ^a^	<0.001
**eGFR (mL/min/1.73 m** ^**2**^ **)**	93.9 (82.1, 105.6)	78.0 (59.1, 88.9) ^b^	66.1 (52.9, 80.5) ^b^	50.1 (27.8, 67.2) ^a^	<0.001
**Serum albumin (mg/dL)**	4.3 (4.2, 4.5)	4.0 (3.7, 4.3) ^b^	4.0 (3.7, 4.2) ^b^	3.7 (3.0, 4.0) ^a^	<0.001
**Serum uric acid (mg/dL)**	4.7 (3.8, 5.9)	5.1 (4.3, 6.1)	5.8 (5.0, 7.0) ^b^	6.7 (5.5, 7.5)^c^	<0.001
**Serum IgA (mg/dL)**	257.5 (225.8, 319.3)	295.0 (217.0, 381.0)	285.0 (215.5, 355.5)	290.0 (240.5, 353.0)	0.425

Data are presented as a median (25^th^, 75^th^ percentiles). BP, blood pressure; eGFR, estimated glomerular filtration rate; TNFR, tumor necrosis factor receptor; UPCR, urine protein-creatinine ratio.

^a^ *P* < 0.05 compared with TNFR1 Q1-3 subgroup in the pairwise comparisons using the Dunn-Bonferroni approach.

^b^ *P* < 0.05 compared with TNFR1 Q1 subgroup in the pairwise comparisons using the Dunn-Bonferroni approach.

^c^ *P* < 0.05 compared with TNFR1 Q1-2 subgroup in the pairwise comparisons using the Dunn-Bonferroni approach.

^d^ *P* value for trend.

**Table 3:** Spearman correlation coefficients between various clinical parameters in IgAN patients.

	UPCR	cTNFR1	cTNFR2	SBP	IgA	Albumin	Uric acid
**eGFR**	-0.32^††^	-0.65^††^	-0.59^††^	-0.17**	-0.07	0.36^††^	-0.49^††^
**UPCR**	1.00	0.49^††^	0.50^††^	0.17**	-0.02	-0.56^††^	0.19**
**cTNFR1**		1.00	0.75^††^	0.14**	-0.02	-0.55^††^	0.52^††^
**cTNFR2**			1.00	0.12*	0.05	-0.53^††^	0.41^††^
**SBP**				1.00	0.11	0.07	0.30^††^
**IgA**					1.00	0.14*	0.14*
**Albumin**						1.00	-0.12*
**Uric acid**							1.00

eGFR, estimated glomerular filtration rate; SBP, systolic blood pressure; TNFR, tumor necrosis factor receptor UPCR, urine protein-creatinine ratio.

* P < 0.05.

** P < 0.01.

^†^ P < 0.001.

^††^ P < 0.0001.

### Histologic features according to the circulating TNFRs

**Fig 2:**
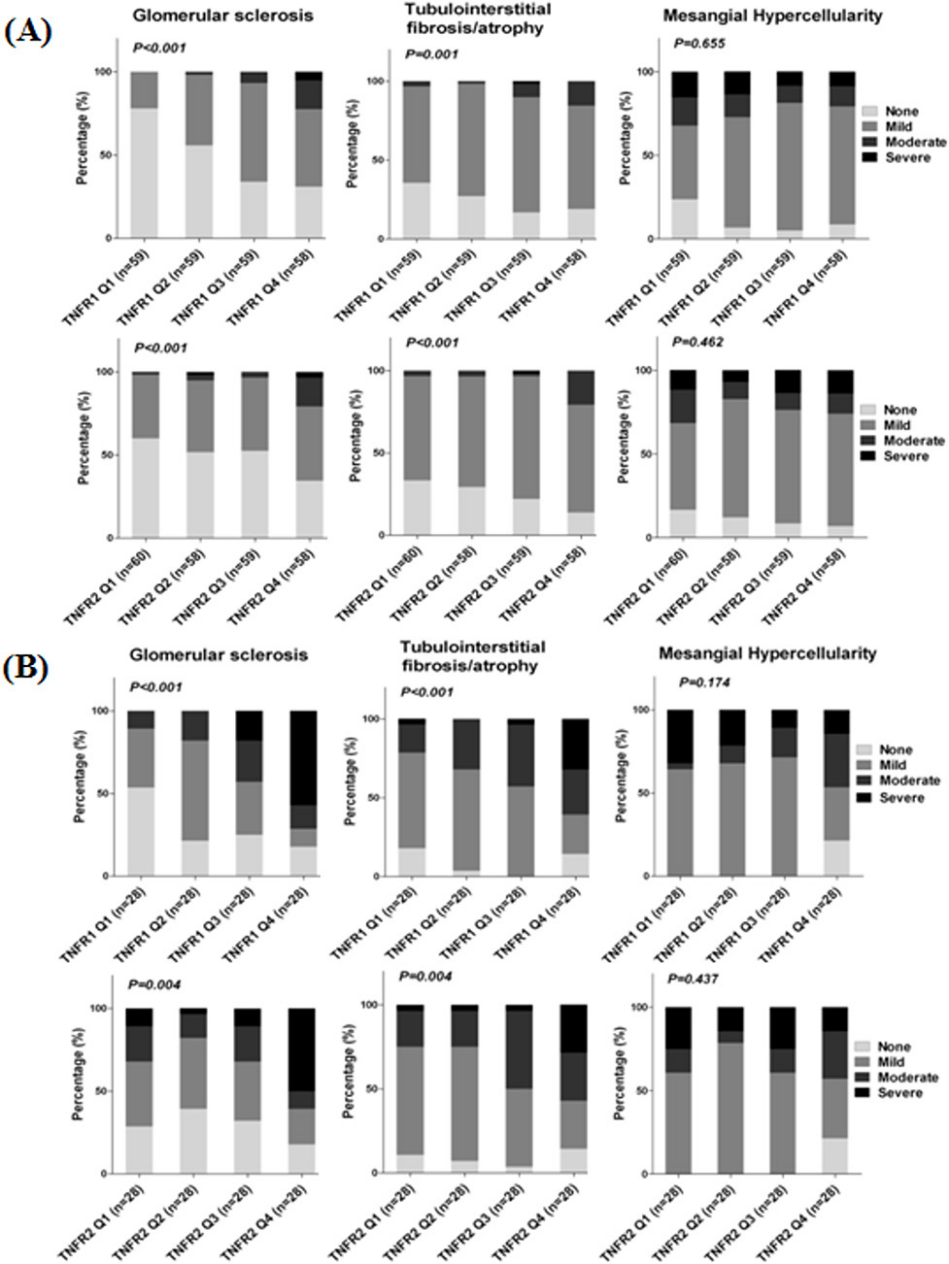
Associations between the concentrations of TNFRs and renal histologic findings. The severity of glomerular sclerosis and interstitial fibrosis/tubular atrophy significantly increased as the concentrations of TNFR1 and TNFR2 increased in both groups with eGFR ≥ 60 mL/min/1.73 m^2^ (A) and with eGFR < 60 mL/min/1.73 m^2^ (B). TNFR, tumor necrosis factor receptors.

We examined the associations between TNFR quartiles at the time of initial diagnosis and the severity of histologic features. To exclude the confounding effect of impaired kidney function on renal histologic changes, stratified analysis based on eGFR of 60 mL/min/1.73 m^2^ was performed. As the concentrations of TNFR1 and TNFR2 increased, the severity of tubulointerstitial lesions such as interstitial fibrosis and tubular atrophy increased gradually in patients with eGFR ≥ 60 mL/min/1.73 m^2^ (Fig 2A) as well as with eGFR < 60 mL/min/1.73 m^2^ (Fig 2B). Among glomerular lesions, only the worsening of glomerular sclerosis was significantly associated with increasing circulating TNFRs in both groups (Fig 2A and 2B). However, the severity of crescent formation was not associated with the concentrations of circulating TNFRs 1 and 2.

### The impacts of circulating TNFRs on clinical outcome

**Fig 3:**
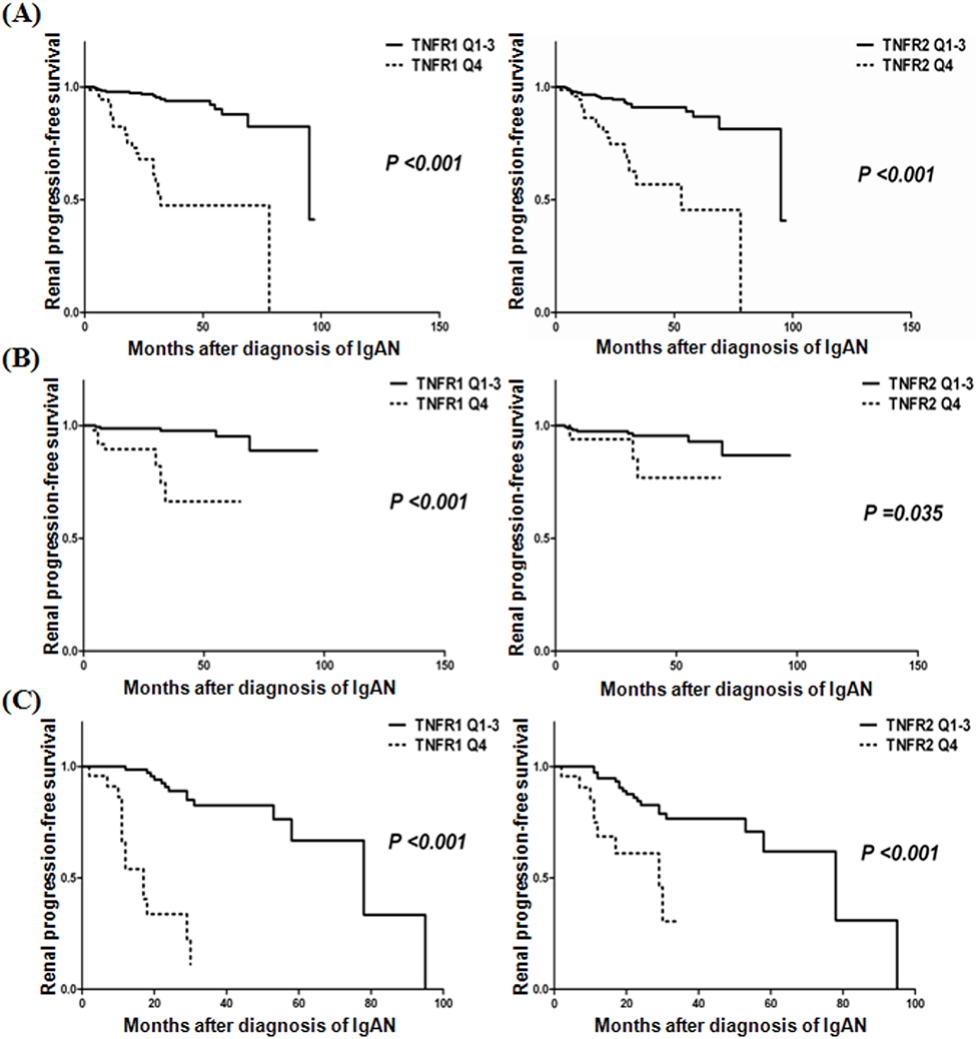
The risk for renal progression according to circulating TNFRs. The highest quartile of TNFR1 and TNFR2 were significantly associated with higher risk for renal progression compared to those in other quartiles of TNFRs, regardless of renal function at the time of diagnosis. (A) Total study population, (B) Patients with eGFR ≥ 60 ml/min/1.73 m^2^, (C) Patients with eGFR < 60 ml/min/1.73 m^2^. eGFR, estimated glomerular filtration rate; TNFR, tumor necrosis factor receptors.

A total of 42 (12.1%) patients reached the primary end point (renal progression; eGFR decline ≥ 30% compared to baseline) during the median follow-up period of 26 months. We investigated the impacts of circulating TNFRs on the renal progression, after stratification of renal function at the time of diagnosis according to eGFR of 60 mL/min/1.73 m^2^ (Fig 3A–3C). The highest quartile of TNFR1 and TNFR2 showed a significantly higher risk for renal progression compared to other quartiles, irrespective of initial renal function. However, the association between the circulating TNFRs and the decline in renal function was more pronounced in patients with initial eGFR < 60 mL/min/1.73 m^2^ than those with eGFR ≥ 60 mL/min/1.73 m^2^.

We also performed the Cox proportional hazards regression analysis (Table 4). Overall, patients in the highest quartile of TNFR1 had a significantly increased risk of renal progression compared with those in other quartiles of TNFR1, after adjustment for age, sex, systolic BP, uPCR, and eGFR (HR 7.48, 95% confidence intervals (CIs) 3.70–15.14, *P* < 0.001). Furthermore, circulating TNFR2 was also an independent predictor of renal progression (HR 2.51, 95% CIs 1.15–5.48, *P* = 0.021). In stratified analysis according to initial renal function classified by eGFR levels of 60 mL/min/1.73 m^2^, elevated concentrations of circulating TNFRs at baseline were significant predictors of renal progression in patients with eGFR ≥ 60 mL/min/1.73 m^2^ as well as eGFR < 60 mL/min/1.73 m^2^.

**Table 4:** Risk factors for renal progression in multivariate Cox regression analysis.

		Total study population			
		**HR** ^**a**^ **(95% CI)**	***P* value**	**HR** ^**b**^ **(95% CI)**	***P* value**
**Age (per 5 years)**		0.99 (0.86–1.14)	0.939	0.97 (0.74–1.26)	0.796
**Male**		1.11 (0.58–2.12)	0.757	0.96 (0.49–1.87)	0.900
**Systolic BP (per 10 mmHg)**		1.07 (0.88–1.31)	0.491	1.08 (0.89–1.32)	0.433
**UPCR (g/g)**		1.22 (1.05–1.43)	0.011	1.24 (1.07–1.45)	0.006
**MDRD-GFR (per 10 mL/min/1.73 m** ^**2**^ **)**		0.98 (0.84–1.15)	0.821	0.85 (0.73–0.99)	0.033
**TNFR1**	**Q1-3 (reference)**	1.00			
	**Q4**	7.48 (3.70–15.14)	<0.001		
**TNFR2**	**Q1-3 (reference)**			1.00	
	**Q4**			2.51 (1.15–5.48)	0.021
		**Patients with eGFR ≥ 60 ml/min/1.73 m** ^**2**^			
		**HR** ^**a**^ **(95% CI)**	***P* value**	**HR** ^**b**^ **(95% CI)**	***P* value**
**Age (per 5 years)**		1.00 (0.79–1.28)	0.977	0.99 (0.78–1.27)	0.954
**Male**		0.70 (0.21–2.29)	0.553	1.22 (0.39–3.79)	0.736
**Systolic BP (per 10 mmHg)**		1.04 (0.71–1.52)	0.828	0.96 (0.66–1.41)	0.851
**UPCR (g/g)**		0.76 (0.46–1.26)	0.285	0.89 (0.53–1.49)	0.655
**MDRD-GFR (per 10 mL/min/1.73 m** ^**2**^ **)**		1.06 (0.99–1.15)	0.102	1.07 (0.99–1.17)	0.095
**TNFR1**	**Q1-3 (reference)**	1.00			
	**Q4**	11.39 (3.26–39.85)	<0.001		
**TNFR2**	**Q1-3 (reference)**			1.00	
	**Q4**			3.25 (1.00–10.54)	0.050
		**Patients with eGFR < 60 ml/min/1.73 m** ^**2**^			
		**HR** ^**a**^ **(95% CI)**	***P* value**	**HR** ^**b**^ **(95% CI)**	***P* value**
**Age (per 5 years)**		0.99 (0.84–1.17)	0.917	0.97 (0.82–1.14)	0.677
**Male**		0.89 (0.37–2.11)	0.785	0.84 (0.38–1.87)	0.670
**Systolic BP (per 10 mmHg)**		1.07 (0.83–1.38)	0.591	1.08 (0.81–1.44)	0.600
**UPCR (g/g)**		1.24 (1.00–1.53)	0.048	1.33 (1.11–1.58)	0.002
**MDRD-GFR (per 10 mL/min/1.73 m** ^**2**^ **)**		1.26 (0.88–1.79)	0.206	0.81 (0.57–1.14)	0.220
**TNFR1**	**Q1-3 (reference)**	1.00			
	**Q4**	10.32 (3.94–27.02)	<0.001		
**TNFR2**	**Q1-3 (reference)**			1.00	
	**Q4**			3.96 (1.60–9.82)	0.003

BP, blood pressure; GFR, glomerular filtration rate; TNFR, tumor necrosis factor receptor UPCR, urine protein-creatinine ratio.

^a^ Clinical parameters were examined together with TNFR1.

^b^ Clinical parameters were examined together with TNFR2.

To determine the effects of IgAN treatment on the predictability of cTNFRs on long-term renal outcome, we also performed the stratified analysis according to the administration of RAS blockers or IS. The patients in the group with the RAS blockers or IS showed greater degree of proteinuria, worse renal function, and higher concentrations of circulating TNFRs 1 and 2 (S1 Table and S2 Table). Circulating TNFRs could predict the renal progression after adjusting for age, gender, and blood pressure, proteinuria, and eGFR measured at the time of kidney biopsy, irrespective of the treatment of RAS blockers or IS. (S3 Table).

**Fig 4:**
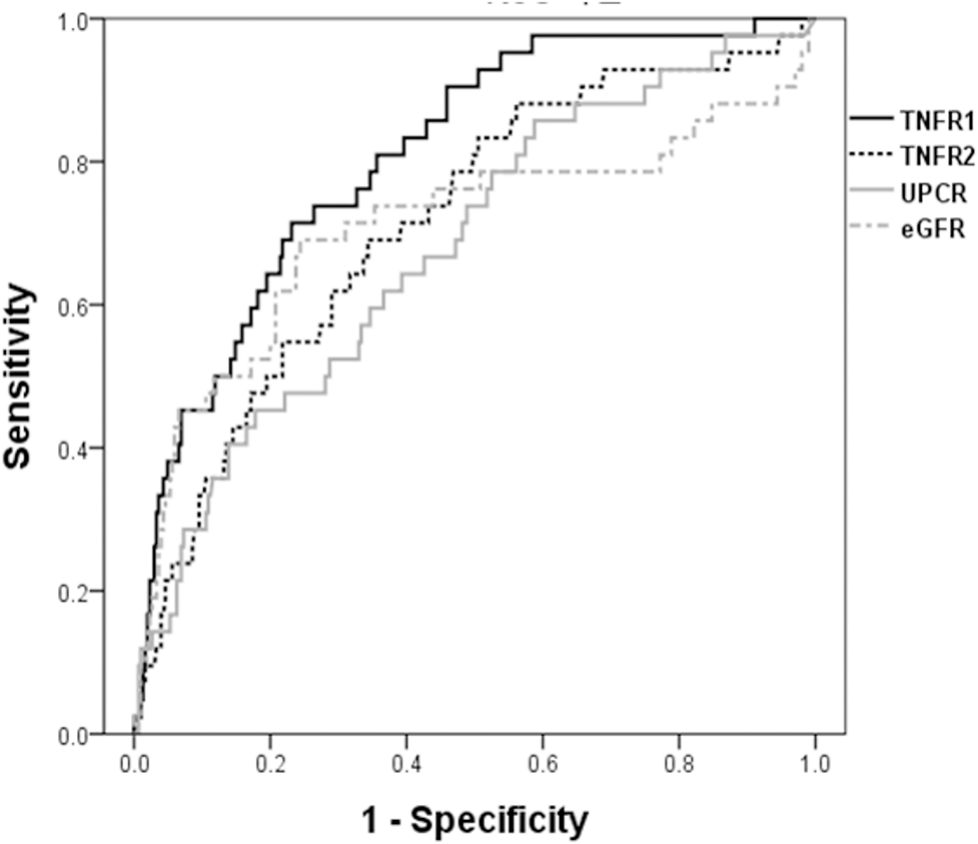
ROC curve analysis with various biomarkers for renal progression. The concentrations of circulating TNFRs can best discriminate patients with renal progression than eGFR or uPCR. eGFR, estimated glomerular filtration rate; TNFR, tumor necrosis factor receptors; UPCR, urine protein creatinine ratio.

Next, we determined whether circulating TNFRs are a better indicator of renal progression than eGFR and uPCR using Receiver operating characteristic (ROC) curve analysis (Fig 4). Circulating TNFRs showed a better performance of predicting renal progression than eGFR or uPCR, showing the largest area under the ROC curves (AUCs); TNFR1, 0.808; TNFR2, 0.714.

## Discussion

This study identified the relationship between circulating TNFRs and various clinical parameters related to kidney damage and the severity of histopathological findings in IgAN. We also demonstrated that elevated concentrations of circulating TNFRs at the time of initial diagnosis were significantly associated with the renal progression in patients with IgAN, regardless of the baseline renal function.

Our findings are consistent with several previous studies, indicating that circulating TNFRs were significantly correlated with the eGFR or proteinuria in patients with various kidney diseases [14–17,26,27]. The changes of uric acid level and serum albumin level following the elevated concentrations of TNFRs in this study were also attributed to the correlation between TNFRs and renal function.

Recently, there have been increasing concerns that TNFRs were strong indicators for deterioration of renal function in various kidney diseases [18,19,28–30]. However, most of prior studies were conducted in diabetic patients or performed cross-sectional analysis only [26,27,31,32]. Although prognostic importance of TNFRs for renal progression in patients with GN has been documented by other studies [22,33], this study was characterized by a different level of significance, given that we determined these findings in Asian patients with IgAN, which are the most common form of GN in Asia. Circulating TNFRs measured at the time of diagnosis predict the renal progression independently of other important risk factors relevant to renal progression in IgAN. Moreover, this predictability of circulating TNFRs on long-term renal outcome was not affected by the treatment of RAS blockers or immunosuppressives. In particular, in stratified analysis according to initial renal function classified by eGFR levels of 60 mL/min/1.73 m^2^ [5,25], the persistent predictive effect of TNFRs even in patients with preserved renal function suggests that the circulating TNFRs at baseline may be early biomarker for renal outcome in IgAN. Higher AUCs of TNFRs compared with uPCR and eGFR for renal outcome in ROC analysis also support the importance of TNFRs as an early biomarker.

Meanwhile, we found more severe tubulointerstitial damage in patients with higher concentrations of circulating TNFRs, irrespective of initial renal function. In other words, higher concentrations of TNFRs may reflect more severe renal histologic damage. It implies that elevated concentrations of circulating TNFRs in the early stages of IgAN may point to advanced renal injury. A previous study on IgAN pathogenesis revealed that mesangial-derived TNFα after IgA deposition activates renal tubular cells, leads to inflammatory changes in the renal interstitium, and eventually tubulointerstitial damage [34,35]. The involvement of circulating TNFRs in tubulointerstitial fibrosis and direct injury to renal tubular cells has also been established [36,37]. Most recently, Sonoda and Gohda, *et al*. demonstrated that elevated cTNFRs were independently associated with the severity of interstitial fibrosis in IgAN patients [38]. Furthermore, TNFRs are involved in chronic inflammation including the release of inflammatory cytokines and up-regulation of vascular adhesion molecules [39–42] and result in apoptosis and cell death in the renal tubules [26]. The essential role of TNFα pathway in pathogenesis of various renal diseases including GN has been documented in earlier clinical and experimental models [39,43–50]. Consequently, such a glomerulotubular cross-talk theory via TNFα pathway activation and proinflammatory reaction may be a clue to explain our findings. Given that tubulointerstitial damage is one of the most important risk factors for renal progression in IgAN [51,52], our findings could be an answer to the question of how elevated concentrations of TNFRs predict the long-term prognosis of IgAN.

There were several limitations to this study. First, the follow-up period is relatively short to assess long-term renal outcomes such as ESRD. Nevertheless, it is noteworthy that this is the first study to indicate a predictive role of circulating TNFRs in a considerably large Asian population with IgAN. To verify long-term consequences of these findings, further studies will be needed. Second, we measured only plasma concentrations of TNFRs. The correlation between plasma and urinary concentrations of TNFRs was demonstrated in an experimental study [53] and circulating TNFRs were shown to be associated with the increased risk of renal dysfunction comparable to urinary TNFRs [18]. Thus, detection of circulating TNFRs alone may be enough to predict renal outcome. In the future, the concentrations of TNFRs in urine or kidney tissue will be measured and analyzed to confirm renal expressions of TNFRs and these impacts.

In conclusion, elevated concentrations of circulating TNFRs at baseline were significantly correlated with poor renal function and advanced renal pathological findings, and were strongly associated with increased risk of renal progression in IgAN. The predictive effect of TNFRs was persistent irrespective of renal function at initial diagnosis. Accordingly, circulating TNFRs can be early biomarkers to predict the severity and clinical outcome in IgAN. Measuring circulating TNFRs may be important and informative in the identification of high-risk patients and in the proper management of IgAN.

## Supporting Information

S1 Table Clinical variables classified according to the administration of renin-angiotensin system (RAS) blockers, (PDF)

S2 Table Clinical variables classified according to the immunosuppressive (IS) treatment. (PDF)

S3 Table Predictability of circulating TNFRs on clinical outcome classified by the immunosuppressive (IS) treatment. (PDF)
