# Supplementary material for: Circulating Tumor Necrosis Factor α Receptors Predict the Outcomes of Human IgA Nephropathy: A Prospective Cohort Study
Source: PLoS One. 2015 Jul 15;10(7):e0132826. doi: 10.1371/journal.pone.0132826 (PMC4503615; doi:10.1371/journal.pone.0132826)
Supplement: S1 Table — (PDF) [file pone.0132826.s001.pdf]

S1 Table. Clinical variables classified according to the administration of renin-angiotensin system (RAS) blockers

	RAS blockers (+) (N = 255, 73.5%)	RAS blockers (-) (N = 92, 26.5%)	Total (N = 347)	*P*
**Age (years)**	39 (31, 49)	30 (21, 43)	37 (29, 48)	<0.001
**Male (n/%)**	111/43.5%	37/40.2%	148/42.7%	0.582
**Smoker (n/%)**	33/12.9%	6/6.5%	39/11.2%	0.095
**Diabetes mellitus (n/%)**	11/4.3%	2/2.2%	13/3.7%	0.354
**Body mass index (kg/m^2)**	23.1 (20.7, 24.9)	21.5 (19.6, 24.2)	22.6 (20.5, 24.8)	<0.001
**Systolic blood pressure (mmHg)**	126 (115, 140)	120 (110, 130)	124 (111, 140)	<0.001
**Microscopic hematuria (n/%)**	228/89.4%	83/90.2%	311/89.6%	0.828
**UPCR (g/g)**	1.06 (0.50, 2.28)	0.57 (0.21, 1.02)	0.91 (0.43, 1.97)	<0.001
**Serum creatinine (mg/dL)**	1.0 (0.8, 1.3)	0.9 (0.8, 1.1)	1.0 (0.8, 1.3)	0.019
**eGFR (mL/min/1.73 m^2)**	70.4 (52.7, 88.1)	82.3 (60.0, 97.2)	74.2 (54.2, 90.8)	0.002
**Serum albumin (mg/dL)**	4.0 (3.7, 4.3)	4.1 (3.7, 4.4)	4.0 (3.7, 4.3)	0.155
**Serum IgA (mg/dL)**	291.0 (234.0, 365.5)	273.0 (173.0, 341.0)	283.0 (222.3, 358.5)	0.005
**Uric acid (mg/dL)**	5.8 (4.6, 7.1)	4.9 (4.2, 6.0)	5.5 (4.4, 6.9)	0.002
**Circulating TNFR1 (pg/mL)**	1148.5 (819.9, 1513.2)	897.0 (597.3, 1339.8)	1077.8 (746.8, 1470.9)	0.001
**Circulating TNFR2 (pg/mL)**	2623.1 (1991.4, 3809.9)	2080.4 (1710.9, 3114.6)	2412.3 (1896.0, 3568.8)	0.002
**Glomerular sclerosis**				0.360
None	108/42.4%	42/45.7%	150/43.2%	
Mild	103/40.4%	36/39.1%	139/40.1%	
Moderate	23/9.0%	11/12.0%	34/9.8%	
Severe	21/8.2%	3/3.3%	24/6.9%	
**Mesangial hypercellularity**				<0.001
None	14/5.5%	18/19.6%	32/9.2%	
Mild	154/60.4%	63/68.5%	217/62.5%	
Moderate	41/16.1%	8/8.7%	49/14.1%	

Severe	46/18.0%	3/3.3%	49/14.1%	
**Interstitial fibrosis/tubular atrophy**				0.124
None	47/18.4%	21/22.8%	68/19.6%	
Mild	155/60.8%	62/67.4%	217/62.5%	
Moderate	43/16.9%	7/7.6%	50/14.4%	
Severe	10/3.9%	2/2.2%	12/3.5%	
**Crescent formation**				0.646
None	199/78.0%	74/80.4%	273/78.7%	
Mild	49/19.2%	17/18.5%	66/19.0%	
Moderate	7/2.7%	1/1.1%	8/2.3%	
**Medical treatment (n/%)**				
Statin	77/30.2%	4/4.3%	81/23.3%	<0.001
Immunosuppressant	36/14.1%	7/7.6%	43/12.4%	0.104

Data are presented as a number (percent) or a median (25th, 75th percentiles).

eGFR, estimated glomerular filtration rate; TNFR, tumor necrosis factor receptor; UPCR, urine protein-creatinine ratio
