# Supplementary material for: Circulating Tumor Necrosis Factor α Receptors Predict the Outcomes of Human IgA Nephropathy: A Prospective Cohort Study
Source: PLoS One. 2015 Jul 15;10(7):e0132826. doi: 10.1371/journal.pone.0132826 (PMC4503615; doi:10.1371/journal.pone.0132826)
Supplement: S2 Table — (PDF) [file pone.0132826.s002.pdf]

S2 Table. Clinical variables classified according to the immunosuppressive (IS) treatment

	IS (+) (N = 43, 12.4%)	IS (-) (N = 304, 87.6%)	Total (N = 347)	*P*
**Age (years)**	43 (30, 50)	36 (28, 48)	37 (29, 48)	0.262
**Male (n/%)**	11/25.6%	137/45.1%	148/42.7%	0.016
**Smoker (n/%)**	5/11.6%	34/11.2%	39/11.2%	0.931
**Diabetes mellitus (n/%)**	3/7.0%	10/3.3%	13/3.7%	0.233
**Body mass index (kg/m^2)**	22.9 (20.4, 24.7)	22.6 (20.5, 24.9)	22.6 (20.5, 24.8)	0.879
**Systolic blood pressure (mmHg)**	122 (110, 140)	124 (114, 140)	124 (111, 140)	0.624
**Microscopic hematuria (n/%)**	38/88.4%	273/89.8%	311/89.6%	0.773
**UPCR (g/g)**	2.85 (1.08, 4.33)	0.79 (0.40, 1.64)	0.91 (0.43, 1.97)	<0.001
**Serum creatinine (mg/dL)**	1.1 (0.9, 1.4)	1.0 (0.8, 1.2)	1.0 (0.8, 1.3)	0.145
**eGFR (mL/min/1.73m^2)**	62.6 (48.1, 76.5)	75.8 (56.0, 92.1)	74.2 (54.2, 90.8)	0.005
**Serum albumin (mg/dL)**	3.6 (3.1, 4.2)	4.1 (3.7, 4.3)	4.0 (3.7, 4.3)	<0.001
**Serum IgA (mg/dL)**	255.5 (220.0, 311.5)	286.0 (223.3, 359.0)	283.0 (222.3, 358.5)	0.299
**Uric acid (mg/dL)**	5.5 (4.4, 7.1)	5.5 (4.4, 6.9)	5.5 (4.4, 6.9)	0.828
**Circulating TNFR1 (pg/mL)**	1393.1 (1077.8, 2195.0)	1026.2 (704.2, 1427.7)	1077.8 (746.8, 1470.9)	<0.001
**Circulating TNFR2 (pg/mL)**	3563.7 (2297.3, 5259.8)	2362.0 (1824.8, 3268.3)	2412.3 (1896.0, 3568.8)	<0.001
**Glomerular sclerosis**				0.060
None	18/41.9%	132/43.4%	150/43.2%	
Mild	13/30.2%	126/41.4%	139/40.1%	
Moderate	9/20.9%	25/8.2%	34/9.8%	
Severe	3/7.0%	21/6.9%	24/6.9%	
**Mesangial hypercellularity**				0.869
None	5/11.6%	27/8.9%	32/9.2%	
Mild	26/60.5%	191/62.8%	217/62.5%	
Moderate	5/11.6%	44/14.5%	49/14.1%	
Severe	7/16.3%	42/13.8%	49/14.1%	

**Interstitial fibrosis/tubular atrophy**				0.774
None	7/16.3%	61/20.1%	68/19.6%	
Mild	26/60.5%	191/62.8%	217/62.5%	
Moderate	8/18.6%	42/13.8%	50/14.4%	
Severe	2/4.7%	10/3.3%	12/3.5%	
**Crescent formation**				0.549
None	33/76.7%	240/78.9%	273/78.7%	
Mild	8/18.6%	58/19.1%	66/19.0%	
Moderate	2/4.7%	6/2.0%	8/2.3%	
**Medical treatment (n/%)**				
Statin	16/37.2%	65/21.4%	81/23.3%	0.022
RAS blocker	36/83.7%	219/72.0%	255/73.5%	0.104

Data are presented as a number (percent) or a median (25th, 75th percentiles).

eGFR, estimated glomerular filtration rate; TNFR, tumor necrosis factor receptor; UPCR, urine protein-creatinine ratio
