# Supplementary material for: Circulating Tumor Necrosis Factor α Receptors Predict the Outcomes of Human IgA Nephropathy: A Prospective Cohort Study
Source: PLoS One. 2015 Jul 15;10(7):e0132826. doi: 10.1371/journal.pone.0132826 (PMC4503615; doi:10.1371/journal.pone.0132826)
Supplement: S3 Table — (PDF) [file pone.0132826.s003.pdf]

S3 Table. Predictability of circulating TNFRs on clinical outcome classified by the immunosuppressive (IS) treatment

	IS (+)	Model 1[a]		Model 2[b]	
		**HR (95% CI)**	***P***	**HR (95% CI)**	***P***
**TNFR1**	**Q1-3 (reference)**	1.00		1.00	
	**Q4**	9.14 (1.85-45.13)	0.007	8.74 (1.77-43.06)	0.008
**TNFR2**	**Q1-3 (reference)**	1.00		1.00	
	**Q4**	7.06 (1.46-34.19)	0.015	6.75 (1.40-32.58)	0.017
	**IS (-)**	**Model 1[a]**		**Model 2[b]**	
		**HR (95% CI)**	***P***	**HR (95% CI)**	***P***
**TNFR1**	**Q1-3 (reference)**	1.00		1.00	
	**Q4**	13.47 (6.09-29.81)	<0.001	9.74 (4.16-22.78)	<0.001
**TNFR2**	**Q1-3 (reference)**	1.00		1.00	
	**Q4**	5.78 (2.82-11.84)	<0.001	3.79 (1.72-8.33)	0.001

[a] Unadjusted model
[b] Model 1 + adjustment for gender, age, systolic blood pressure, eGFR, urine protein-creatinine ratio, and the quartiles of concentrations of cTNFRs

TNFR, tumor necrosis factor receptor
